# Radial glia and radial glia-like cells: Their role in neurogenesis and regeneration

**DOI:** 10.3389/fnins.2022.1006037

**Published:** 2022-11-16

**Authors:** Yamil Miranda-Negrón, José E. García-Arrarás

**Affiliations:** Department of Biology, College of Natural Sciences, University of Puerto Rico, San Juan, Puerto Rico

**Keywords:** regeneration, echinoderm, spinal cord, radial nerve cord, radial glia (RG), radial glia like cells

## Abstract

Radial glia is a cell type traditionally associated with the developing nervous system, particularly with the formation of cortical layers in the mammalian brain. Nonetheless, some of these cells, or closely related types, called radial glia-like cells are found in adult central nervous system structures, functioning as neurogenic progenitors in normal homeostatic maintenance and in response to injury. The heterogeneity of radial glia-like cells is nowadays being probed with molecular tools, primarily by the expression of specific genes that define cell types. Similar markers have identified radial glia-like cells in the nervous system of non-vertebrate organisms. In this review, we focus on adult radial glia-like cells in neurogenic processes during homeostasis and in response to injury. We highlight our results using a non-vertebrate model system, the echinoderm *Holothuria glaberrima* where we have described a radial glia-like cell that plays a prominent role in the regeneration of the holothurian central nervous system.

## Origin and description of radial glia cell

**FIGURE 1:**
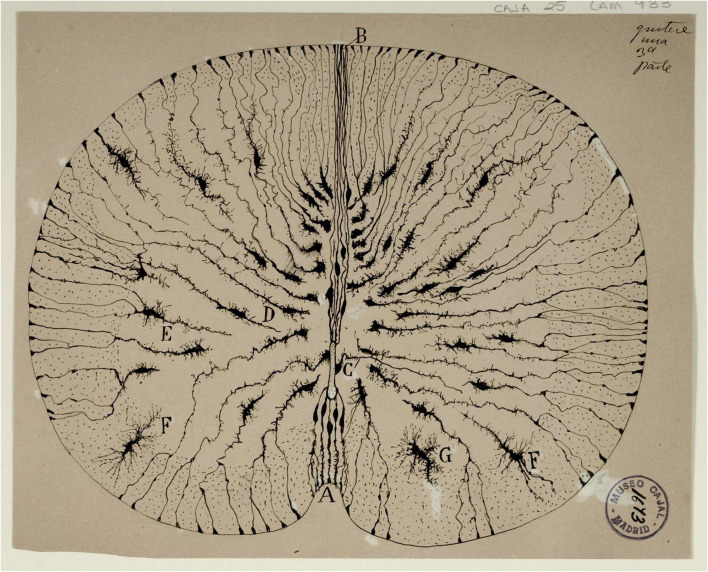
Original drawing by Santiago Ramón y Cajal depicting the radial glia cells in Golgi-stained neonatal mouse spinal cord sections. The radial morphology of the cells, extending from the central canal to the outside perimeter of the cord is clearly observed. Reproduced with permission from the Cajal Institute, Madrid.

Radial glial cells (RGCs) were initially described around the mid-late 1800s in several mammalian species by Bentivoglio and Mazzarello (1999). Using the Golgi impregnation technique, these and other investigators reported that RGCs were found in the developing nervous system (NS) at different embryonic and neonatal stages (Figure 1). Ever since then, RGCs have been associated with: (1) characteristic morphological features, (2) their occurrence in nervous tissue, specifically in the central nervous system (CNS), and (3) their transient presence during embryonic development. Over one century later, multiple studies have confirmed the original findings and have extended them to incorporate the molecular basis of cellular phenotypes and their functions. RGCs, or cells with similar properties termed radial glia-like cells (RGLCs), have been documented in adults of various species, where functions, additional to their embryological role, have been described. In this review, we focus on the presence of RGLC’s in adult organisms and their roles in NS regeneration, we highlight the work of our group in studying the RGLCs in echinoderms and their prominent role during regeneration of the radial nerve cord (RNC).

### Radial glia in vertebrates

**FIGURE 2:**
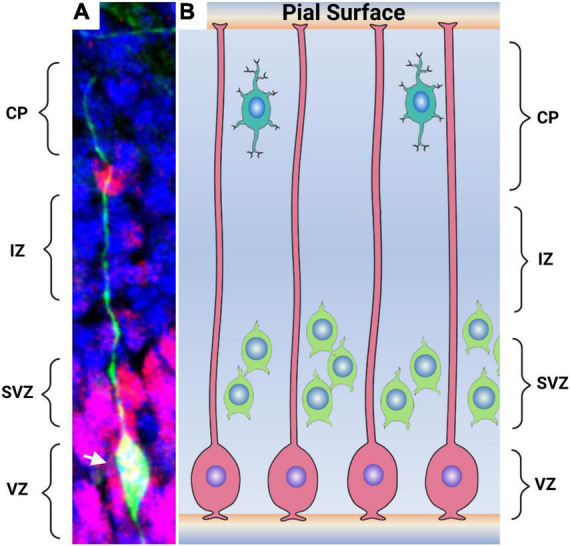
Radial glial cells in the embryonic mammalian brain. **(A)** The morphology of a mammalian glia cells are shown by the expression of GFP. Radial glia cell processes extending from the ventricular surface to the pial surface of the brain are distinguishable. The cell body (arrowhead) lies within the ventricular zone. Non-radial pluripotent cells are seen by Pax6 in red while the remaining cell are shown in blue stain by DAPI. **(B)** An artistic representation of the radial glia cells (pink) in the developing brain with processes extending from the ventricular surface to the pial surface of the brain. Intermediate precursors and neuronal precursors (green) derived from the radial glia are observed in the subventricular zone. Differentiated neurons (aqua) located close to the pial surface. CP-cortical plate, IZ-intermediate zone, SVZ-subventricular zone, and VZ- ventricular zone. Image 2A courtesy of Joseph Elsbernd.

Magini’s original description of RGCs consisted solely of the cell’s morphological features and the changes they displayed during vertebrate NS development. In one of his Golgi-stained sections, Bentivoglio and Mazzarello (1999) describes them as “very long and thin filaments, mostly directed toward the white matter, where they often get lost after having traversed the entire thickness of the gray matter”. This description, with emphasis on the apico-basal radial morphology, has been substantiated using many other techniques. Among them immunohistochemical and transgenic tools (Figure 2). Many of these tools have also served to study the cell’s origin.

Radial glial cells originate from the early neural tube neuroepithelial cells (NECs) by a transition that involves acquiring astroglia characteristics. NECs are known for their apico-basal polarity and interkinetic nuclear migration associated with their cell cycle progression. At the beginning of neural tube formation, NECs divide symmetrically, populating and supporting the development of the neural tube. Eventually, most NECs will differentiate into RGCs. At this point, gene expression changes are observed, and unique markers for the RGCs are expressed. For example, tight junction occludins are replaced by cell adhesion proteins such as N-cadherins (Aaku-Saraste et al., 1996). Similarly, cytoplasmic glycogen granules begin to appear (Gadisseux and Evrard, 1985). Other changes can be seen at the transcriptional level, where the involvement of repressor-type basic helix-loop-helix (bHLH) gene Hes3 is downregulated while Hes5 expression increases (Hatakeyama et al., 2004; Kageyama et al., 2008). RGC progenitor cells will divide asymmetrically, self-renewing, giving rise to progenitor cells and eventually differentiating into neuronal and glial cells (Haubensak et al., 2004; Noctor et al., 2004; Rowitch and Kriegstein, 2010).

Radial glial cells identification has evolved from recognition solely based on morphology to the identification of RGC markers and, more recently, RGC gene expression profiles. In these studies, it has been shown that RGCs change their morphology and gene expression during the developmental process. Thus, the description of their gene expression profile and/or their specific marker expression has been used to define RGC subpopulations that are found at specific developmental stages or in certain regions of the CNS.

Historically, at the gene expression level, cells that expressed glutamate aspartate transporter (GLAST) (*SCL1A3*), glutamate synthase (GS), S100β, tenascin C (TN-C), brain lipid binding protein (BLBP), SCO-spondin, and the intermediate filaments glial fibrillary acidic protein (GFAP), Nestin, and Vimentin (VIM) were generally considered RGCs (Mori et al., 2005; März et al., 2010; Diotel et al., 2020). Therefore, these markers have been adopted as the “typical” RGC markers. However, not all of them are RGC specific. For example, BLBP, S100β, GFAP, and GS are also expressed by NECs and some astrocytes (Robel et al., 2011; Lattke and Guillemot, 2022). Moreover, some of these markers are only expressed transiently in RGCs, depending on the differentiation stage (Gotz et al., 2013). In view of these provisos, the repertory of RGC markers is continuously expanding with the discovery of new gene products. Among these are Sox2, PAX6, Hes1, Hes5, and N-cadherin, that are considered markers that can be of use to identify RGC populations in rodents and humans (Levitt and Rakic, 1980; Kageyama et al., 2008; Malatesta et al., 2008; März et al., 2010; Krencik and Zhang, 2011; Johnson et al., 2016). In the last few years, our knowledge of RGCs has expanded significantly due to the use of single-cell RNA-sequencing (sc-RNAseq) technology. Additional putative markers for RGCs have been proposed, such as PDGFD, HOPX, and ITGB5, identified in human stem cells of the ventricular and outer subventricular neocortex. These markers still need to be further characterized to determine their specificity to RGCs (Pollen et al., 2015).

Differences between cell cycle, markers, and neuronal fate have made it clear that RGCs comprise a heterogenic population (Pinto and Götz, 2007; Malatesta et al., 2008; Pollen et al., 2015; Scott et al., 2021). This was well established in the early 2000 when colocalization analysis reveal different RGC marker expressions within the RGCs (Hartfuss et al., 2001). In both the zebrafish and human CNS, studies have shown that RGCs are more heterogenic than originally thought. For example, sc-RNAseq experiment of the zebrafish spinal cord revealed a small subset of RGCs to be oligodendrocyte progenitor cells given their Sox19a and olig2 marker expression (Scott et al., 2021). Similarly, in the human telencephalon, nine different RGC progenitor clusters were identified via a combination of VIM, SOX2, Hes5, and DLK1 gene expression profiling and immunostaining (Eze et al., 2021). Human cortical RGC also showed a transcriptional diversity that apical versus non-apical RGCs which were then classified as pro-neural or multipotent RGC (Johnson et al., 2015). It is expected that the growing identification of markers increases the likelihood of presenting a comprehensive characterization of RGC origin, development, and possible differences between species, brain regions, and/or developmental stages.

As is evident, the remaining challenge is to characterize cellular populations that might differ among developmental stages, anatomical regions and/or species in their gene expression, to determine developmental, evolutionary and functional homologies. In Table 1 we have listed some markers commonly used to identify RGC and RGLC and the species where they have been detected.

**TABLE 1:** Radial glia and radial glia-like cell markers.

	Rodents				Zebrafish			
Gene/Marker/Antigen	Embryonic	Adult SGZ	Adult SVZ	Müller glia	Larvae	Adult VZ	Müller Glia	Axolotl
Radial morphology	+	+/−	+	+	+	+	+	ND
BLBP	+	+	+	−	−	+	−	ND
Cyp19a1b (aromatase B)	−	−	−	−	+	+	−	ND
GFAP	−	+	+	+	+	+	+	+
GLAST	+	+	+	+	ND	ND	+	ND
GS	+	ND	ND	+	ND	+	+	ND
Her4.1	−	−	−	−	−	+	−	ND
Hes1	+	+	+	+	−	−	−	ND
Hes5	+	+	+	+	−	−	−	ND
Nestin	+	+	+	ND	+	+	ND	+*
Pax6	+	ND	+	+*	−	−	+	+
S100B	+	−	−	ND	ND	+	ND	ND
Sox2	+	+	+	+	+	+	+	+
Tenascin−C	+	ND	+	ND	ND	ND	ND	ND
Vimentin	+	ND	+	+	+	+	+	+*

+*, marker is expressed only in response to injury. +/−, two types of glia are found in this region. One with radial morphology and another non-radial. ND, no information regarding marker was found in these cells. The table illustrates radial glia markers’ presence during development and radial glia-like cells of adults in different cerebral regions. It takes into consideration markers found for rodents, zebrafish, and axolotl’s spinal cord. Table was created using the following references: Holder et al. (1990), Akimoto et al. (1993), Walder et al. (2003), Bernardos et al. (2007), Kageyama et al. (2008), Thummel et al. (2008); März et al. (2010), Hutton and Pevny (2011), Gotz et al. (2013), Sundholm-Peters et al. (2004), Patro et al. (2015), and Tazaki et al. (2017), among others already cited in this article.

### Radial glia-like cells in vertebrates

Due to their initial association with embryological development, the term RGC has not been well accepted to name cells that express radial morphology and RGC markers in the adult vertebrate CNS. The nomenclature used to name these cells mostly depends on the species or on the anatomical region where they are found. For example, radial ependymoglial cells, Bergmann radial glia, and Müller glial cells are terms used to describe them in the spinal cord, cerebellum, and retina, respectively (Goldman, 2014; Gao et al., 2021; Walker and Echeverri, 2022). Similarly, cells that share these characteristics in the subventricular zone (SVZ) and subgranular zone (SGZ) of adult mammals have been interchangeably addressed in the literature as neural stem cells (NSC), RGLC, and radial astrocyte (RA) (Gebara et al., 2016; Berg et al., 2018; Joven and Simon, 2018; Lust and Tanaka, 2019; Obernier and Alvarez-Buylla, 2019; Urbán et al., 2019; Zambusi and Ninkovic, 2020). Given their original description as a glial cell linage, in this review we will use the term RGLC when referring to these adult cells, while the term RGC will be reserved to name the embryonic cells.

**FIGURE 3:**
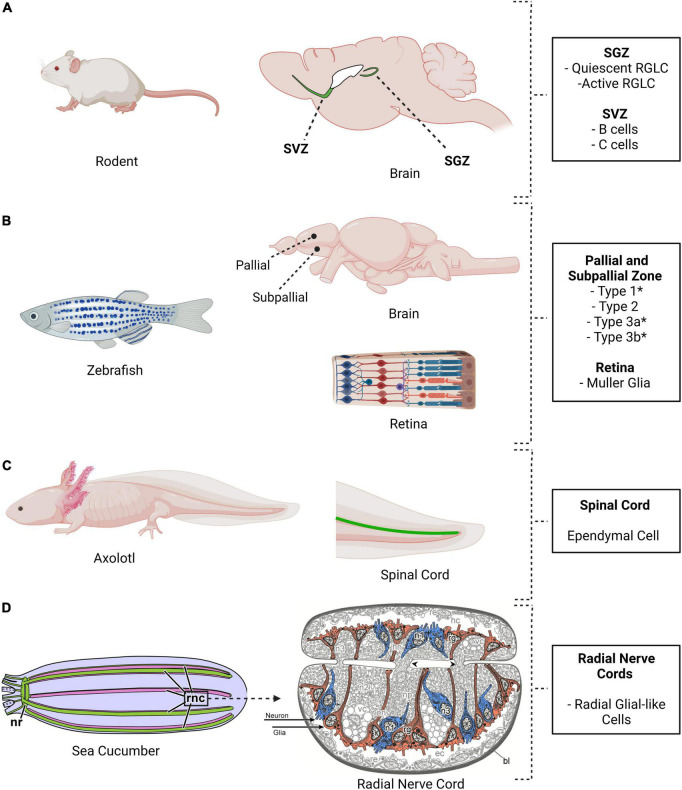
Location of radial glia cells and radial glia-like cells in four different model systems. **(A)** Mouse, **(B)** Zebrafish, **(C)** Axolotl, and **(D)** Sea cucumber. These species share the presence of cells with neurogenic properties. The adult mouse brain shows two major niches, the subventricular zone (SVZ) and the subgranular zone (SGZ). Radial glia-like cells are present and neurogenesis takes place in these niches. Multiple areas in the adult zebrafish brain are neurogenic, as are the Müller glia in the retina. **(C)** Ependymal cells, a type of radial glia-like cells, are also present in the axolotl spinal. **(D)** Radial glial-like cells are found in the holothurian nervous system. *H. glaberrima* nervous system, like that of other echinoderms, comprises an anterior nerve ring (nr) and five radial nerve cords (rnc) that runs from anterior to posterior. The radial nerve cords are ganglionated with the neuronal cell bodies (blue) and the glial cell bodies (red) in the periphery of the neuropile (Created using biorender and illustrations adapted from Mashanov et al., 2015c).

The best case to consider RGLCs in adult vertebrates is probably the existence of cells that continue to proliferate throughout the animal’s life in the hippocampal dentate gyrus (DG) and in the SVZ of the lateral ventricle (Götz and Barde, 2005; Urbán et al., 2016, 2019; Obernier and Alvarez-Buylla, 2019; Figure 3). Those that reside in the SGZ have long processes that reach the granule cell layer and extend into the inner molecular layer. Extensive studies on this region have categorized the RGLC population into type I and type II cells also known as quiescent NSC (qNSC) and active NSC (aNSC) (Gonçalves et al., 2016; Urbán et al., 2016). Besides their morphological resemblance to embryonic RGCs, they also share distinctive marker expressions, such as BLBP, Sox2, GFAP, and Nestin (Bonaguidi et al., 2011).

Similar to what has been observed in the SGZ, the adult SVZ niche also contains cells expressing astroglia markers and having radial glial-like morphology. These cells, called B cells, were initially thought to be proliferating astrocytes given their astroglial marker expression (Doetsch et al., 1999; Capilla-Gonzalez et al., 2015). However, they were eventually demonstrated to be neurogenic progenitors, resembling types I and II RGLCs of the SGZ niche (Rushing and Ihrie, 2016). Due to their progenitor capacity, they are also commonly addressed in the literature as NSC, while others have used the term “radial astrocytes” or “SVZ astrocyte” (Kriegstein and Alvarez-Buylla, 2009). The B cells also share with SGZ cells their distinction by proliferative capacity, where qNSC give rise to aNSCs and differ in some of the markers that they express (Berg et al., 2018; Ruddy and Morshead, 2018). For example, upon activation, quiescent B cells upregulate Nestin and EGFR (Ruddy and Morshead, 2018).

Radial glia-like cells have also been extensively studied in the adult zebrafish brain, mainly in the telencephalon but also in the hindbrain, mesencephalon, cerebellum, medulla oblongata, and retina (Grandel et al., 2006; Than-Trong and Bally-Cuif, 2015; Barbosa and Ninkovic, 2016; Diotel et al., 2020; Figure 3). The best studied region is the pallial zone, located in the dorsal telencephalon. Given that this region is on the brain’s outer surface, it is more accessible for experimental procedures. It has been documented that some of the residing cells within this region bear a morphology reminiscent of mammalian RGCs (Barbosa and Ninkovic, 2016). They show a polarized morphology with their bodies within the ventricular surface and their processes spanning through the parenchyma. Cells in the pallial region express GFAP, BLBP, Fabp7a, Sox2, Her4.1, Hey1, and Nestin, meeting the accepted distinctive RGC molecular profile (Barbosa et al., 2004; Dray et al., 2015; Than-Trong et al., 2018). Heterogeneity between cells in the pallial niche has also been proven, classifying them as type I and type II cells based on their proliferative status (März et al., 2010). Analogous to adult rodent cells in the SGZ, most RGLCs in the zebrafish are in a quiescent state (known as type I cells) while only 5% are actively proliferating (known as type II cells) as shown by PCNA or MCM2/5 expression (Adolf et al., 2006; Diotel et al., 2010, 2020; Rodriguez Viales et al., 2015; Labusch et al., 2020).

In summary, the present evidence strongly suggests that an RGC-like population persists through vertebrate development and can be found in certain specialized niches in the adult brain.

### Radial glia in invertebrates

While RGCs have been described in all vertebrate classes, establishing the occurrence of RGC in invertebrates has been a challenge. Studies of radial glia in invertebrates can be described, at their best as patchy, where some animal groups have been studied while others have been largely ignored. These studies also suffer from differences in the use of scientific tools or markers to characterize the cells. Nonetheless, some bona fide RGLCs have been described in invertebrate organisms, and most of these reports can be found in the review of glia evolution authored by Verkhratsky et al. (2019).

Probably, the most extensive comparative study of RGLCs in invertebrates was done using transmission electron microscopy (TEM) and immunolabeling for SCO-spondin glycoprotein (Helm et al., 2017). Researchers analyzed two invertebrate protostomes (the annelid *Owenia fusiformis* and the priapulid worm *Pirapulus caudatus)* and two invertebrate deuterostomes, the non-chordate sea star *Asteria rubens* and the hemichordate acorn worm *Balanoglossus misakiensis.* They reported cells with prominent radial morphology in all species. The radial morphology correlated with the presence of immunoreactivity to Reissner’s substance (the major component of Reissner’s substance is SCO-spondin). Although positive immunoreactivity was observed in all organisms, extensive data was provided only for *O. fusiformis*, where positive labeling for RGLCs was found in larval, juvenile, and adult animals (Helm et al., 2017). Thus, RGLCs expressing at least one RGC marker were found in protostomes. A different study, focusing on another annelid, the earthworm *Eisenia fetida* identified two types of cells: the long process glial cells (named neurilemmal glial cells) in the ventral ganglion periphery and another glial cell (supporting-nutrifying cells) (Csoknya et al., 2012). However, in this case, they reported positive GFAP immunoreactivity in the supporting cells and not in the cells with radial morphology. Thus, showing some of the inconsistencies that plague the protostome reports.

In contrast to the invertebrate protostomes, RGLCs have been reported in the three major groups of invertebrate deuterostomes: the Echinodermata, the Hemichordata and the non-vertebrate Chordata. In fact, some investigators propose that it is within the deuterostomes that RGCs first appeared and undertake a pivotal role in the formation of the CNS (Verkhratsky et al., 2019). For example, RGLCs have been identified in the Amphioxus CNS (Bozzo et al., 2021). Amphioxus is a cephalochordate (a subphylum that groups non-vertebrate considered the closest relatives of the Vertebrata). One study analyzed the expression of RGC markers (EAAT2, GFAP/vimentin, and SCO-spondin) by cells in the developing CNS, asserting that some of these markers are expressed by cells with radial morphology (Bozzo et al., 2021). Moreover, these embryonic cells are the progenitors of RGLCs found in the adult lancelet CNS in regions that are known to sustain neurogenesis (Bozzo et al., 2021).

**FIGURE 4:**
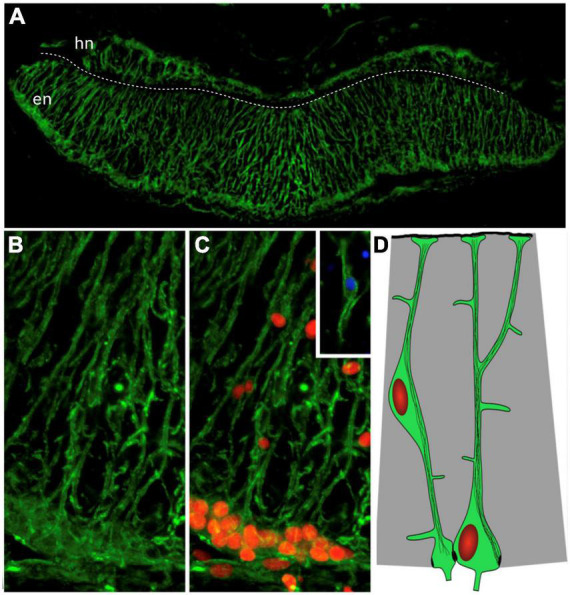
Radial glia-like cells in the holothurian radial nerve cord. **(A)** Cross section of the radial nerve cord of the sea cucumber *H. glaberrima* showing immunoreactivity to ERG1 antibody. This antibody recognizes the radial glia-like cells full extension from the apical to the basal borders. **(B,C)** Magnification of the ectoneural band highlighting the radial morphology of the cell extensions and the presence of the nuclei (red) within the cell bodies. Insert shows the cell body of a glial cell found within the neuropile. The white dash line divides the hyponeural region (hn) from the ectoneural region (en) of the radial nerve cord. **(D)** Diagram depicting the cell body and extensions with cytoskeletal filaments (Adapted from Mashanov et al., 2010).

There is also strong experimental support for the presence of RGLCs in the echinoderms (Mashanov and Zueva, 2020). Studies performed by our group in the sea cucumber *Holothuria glaberrima* have identified RGLCs by employing a combination of cell morphology, Reissner’s substance immunolabeling, and the expression of a yet to be characterized antigen recognized by monoclonal antibodies (Mashanov et al., 2010, 2013). The RGLCs were described in radial nerve cords (RNCs), which are ganglionated nerves that, like the vertebrate spinal cord, contain neuronal and glial cell bodies and extensive fiber tracts. In the echinoderms, the neuronal and glial cell bodies are localized toward the periphery of the cords, while the fibers are mainly found in a neuropile in the center (Figures 3, 4). In *H. glaberrima*, RGLCs represent 60–70% of the cells in the RNCs consisting of most, if not all, glial cells present (Mashanov et al., 2010; Mashanov and Zueva, 2020). Mashanov et al. (2015a) outlined the general similarities of the echinoderm RGLC and vertebrate RGCs in their published chapter on the NS of the Echinodermata*:* “(i) *orthogonal orientation of the cell’s main axis to the plane of the neuroepithelium;* (ii) *highly elongated shape of the cells allowing them to span the entire thickness of the neuroepithelium between the apical and basal surfaces*, (iii) *long thick bundles of intermediate filaments, which run mostly along the main axis of the cell and fill almost the entire intracellular space of the glial processes*, (iv) *short protrusions branching off at a right angles from the main processes and penetrating into the surrounding neural parenchyma”* and (v) the expression of a *“material similar to the so-called Reissner’s substance of chordates”*.

The holothurian RGLCs constantly proliferate in the adult sea cucumber RNC lateral regions (Mashanov et al., 2005). However, it is still unknown whether these cells proliferate symmetrically or asymmetrically, nor the type of cells produced under homeostatic conditions (Mashanov et al., 2015c). Differential expression patterns were documented by *in situ* hybridization studies identifying *Myc* transcripts, suggesting that RGLCs are a heterogeneous population. Nonetheless, additional validations are required for a more in-depth characterization of RGLC subpopulations.

In summary, cells with radial glial morphology have been described in several invertebrate groups, however, in many cases, the typical RGC vertebrate molecular markers are not documented. Most of the early studies identifying these cells in invertebrates focus on their radial morphology but are hindered by the possible limited cross-reactivity of antibodies that recognize vertebrate RGC markers when used against invertebrate tissues. Thus, identifying their presence in invertebrates remains an enduring process. However, the availability of transcriptome studies promises to advance the comparative studies needed to characterize invertebrate RGLCs. Future experiments using new and evolving techniques will improve invertebrate RGLC identification and serve to elucidate their relationship to the vertebrate RGC and RGLC populations (Csoknya et al., 2012; Verkhratsky et al., 2019).

## Roles and functions of radial glia cells

### Their defining developmental role

**FIGURE 5:**
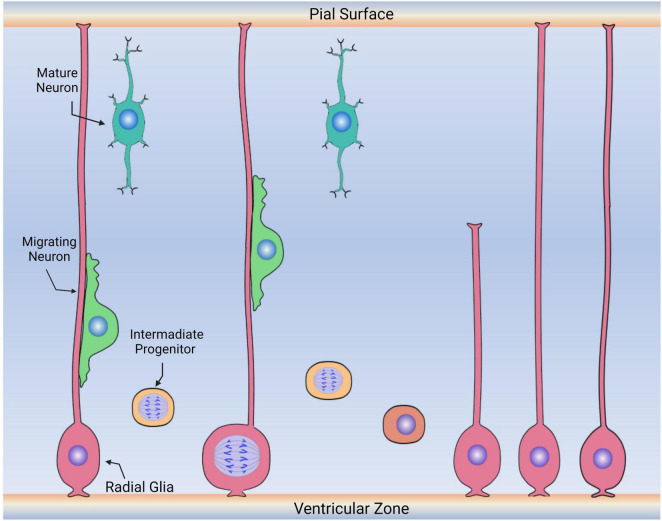
Diagram depicting the embryonic roles of radial glia cells. During embryonic development, radial glia cells (pink) divide symmetrically and asymmetrically, generating other radial glia and intermediate progenitors (labeled in yellow) that will continue to migrate, proliferate and differentiate into new neurons or glia. Radial glia cells also serve as the scaffold for the migration of neuronal precursors (green) to the upper layers of the cerebral cortex. Following migration, these cells then differentiate into neurons (aqua) or other glia (not shown).

The roles of RGCs were first described in mammals during embryonic development. The original descriptions and putative roles were based on histological and anatomical data and, as such, were subject to different interpretations (Bentivoglio and Mazzarello, 1999). RGCs were easily identified at different developmental stages by their elongated fibers that traversed the neural tube from the ventricular zone, where the cell bodies are located, to the pial surface (Figure 5). The functions of these morphological features were elucidated in the early 1970s by Pasko Rakic in a series of trailblazing experiments (Rakic, 1971; Bentivoglio and Mazzarello, 1999; Shu and Richards, 2001; Barresi et al., 2005; Johnson et al., 2016). Using electron microscopy, he and colleagues showed that in rhesus monkeys, RGCs were responsible for guiding and distributing migrating newborn neurons from the ventricular zone to the developing cortex. Thus, they proposed that RGCs function as scaffolding cells for migrating cerebral cortex neurons. In addition to the scaffolding functions, RGCs were subsequently found to divide and eventually give rise to many brain components. These include not only cells but functional structures, such as their interaction with endothelial cells to form what will become the blood-brain barrier (BBB) (Johnson et al., 2016).

In the developing mammalian brain, RGCs divide symmetrically and asymmetrically, self-renewing, and giving rise to other cell populations (Figure 5; Haubensak et al., 2004; Noctor et al., 2004). Some of these cells are neurons, while others are ependymal cells, glial cells (astrocytes and oligodendrocytes), and stem cells (here considered RGLCs) that will continue to reside in specific niches of the adult brain (Rakic, 1995; Malatesta et al., 2003; Merkle et al., 2004; Spassky et al., 2005). The RGC behavior is greatly influenced by intrinsic cell signaling and local environmental signals (Götz et al., 2002). It is now accepted that RGCs consist of a heterogenous population of progenitor cells, some with a predetermined capacity to differentiate into neurons (Malatesta et al., 2000, 2008; Howard et al., 2008). Other populations are more prone to give rise to glial cells. For example, at the end of the human embryonic developmental period, approximately 20 weeks after fertilization, some RGCs begin their transformation into GFAP^+^ astrocytes, losing their ventricular contacts while acquiring the astrocytic morphology (deAzevedo et al., 2003; Howard et al., 2008). Thus, a relationship between RGC disappearance and astrocyte number increase has been demonstrated by cell birth lineage tracing experiments (Voigt, 1989).

### Roles of vertebrate radial glia-like cells

The main role associated with all RGLCs is homeostatic neurogenesis. Neurogenesis is the birth of new neurons via precursor cells (intermediate progenitors and neuroblasts) that originate from RGLC proliferation and maturation (Encinas et al., 2011; Berg et al., 2018). Although neurogenesis is mainly associated with embryonic development, some animals maintain a homeostatic neurogenic process throughout most, if not all, their life span (Lust and Tanaka, 2019; Abbott and Nigussie, 2020; Zambusi and Ninkovic, 2020). In fact, one of the earliest examples of adult neurogenesis was reported to occur during the breeding season in the CNS of adult canaries (Nottebohm, 1989). In adult rodents, RGLCs of the SGZ have been shown to produce excitatory neurons that might be involved in memory formation and learning capacity (Ming and Song, 2011; Berg et al., 2018). Similarly, the RGLC of the SVZ are associated with adult neurogenesis. But in this case with the formation of inhibitory neurons that contribute to odor discrimination and odor-reward association (Grelat et al., 2018; Li et al., 2018). It is important to consider that, despite their proliferative capacities, most of these cells are in a dormant or quiescent state (Bonaguidi et al., 2011; Berg et al., 2018). In fact, the study of quiescence and proliferation of RGCLs has become an important area of study (Urbán et al., 2016, 2019; Lattke and Guillemot, 2022). It is believed that the quiescent state is necessary to conserve stemness, therefore maintaining the cell’s neurogenic potential. The hypothesis being, that this cell state avoids DNA, protein, or mitochondrial damage that might lead to premature cell senescence, eliminating the stem cell niche (Urbán et al., 2019; Labusch et al., 2020). Particular attention has been placed on identifying the local niche signals that control quiescence, such as the Notch pathway or ASCL1 (Geribaldi-Doldán et al., 2018; Harris and Guillemot, 2019; Dray et al., 2021; Harris et al., 2021; Terreros-Roncal et al., 2021). Other experiments are focused on signaling pathways that channel the newly generated cells toward a neuronal versus a glial phenotype (Domínguez-García et al., 2020, 2021).

In the adult zebrafish brain, sixteen RCLC niches provide the organism with lifelong neuron replacement sites (Labusch et al., 2020). In the telencephalon, cell types I, II, IIIa, and IIIb have been suggested to be the main neurogenic cells (März et al., 2010; Diotel et al., 2020). Type I and II cells have been characterized as quiescent and proliferative, respectively, while type IIIa/b are thought to be higher committed progenitor cells (Diotel et al., 2020). These classifications highlight the similar roles of RGLCs in zebrafish and rodents, where quiescent cells maintain niche and stemness while active cells replenish the tissue with new neurons. Breakthroughs in single-cell transcriptomic sequencing have provided important information on RGLC progeny and cell heterogeneity. In particular, experiments performed with transgenic zebrafish have shown that the RGLC population is able to generate a diverse pool of newborn neurons (Lange et al., 2020). Moreover, some of these RGLC also expressed olig2 marker suggesting the presence of a RGLC subpopulation that will transition to oligodendrocyte progenitors (Lange et al., 2020). Thus, these studies demonstrate the heterogeneity of the RGLC population in the vertebrate brain.

In summary, RGLCs in adult organisms appear to be associated with the formation of new neurons. The present evidence strongly suggests that an RGLC population persists through vertebrate development and is involved in neurogenesis in the adult brain. The limitations in neurogenesis, or its absence in most regions of the adult vertebrate brain and spinal cord, account for the limited regeneration abilities of the vertebrate CNS. Therefore, the second part of this review will focus on the role of RGLCs in NS regenerative processes. We begin this section with an overview of NS regeneration, the models where it has been studied, and the mechanisms that take place.

## Principles of nervous system regeneration

Nervous system regeneration, or the lack of it, remains one of the least understood and most diverse areas of study in the field of regeneration biology. Many species can regenerate their NS components, though the cellular and molecular machinery they employ to attain their regeneration capacities can differ. Hence, understanding the fundamental biological processes by which regeneration-competent species achieve regeneration has been the field’s holy grail for quite some time. Given the diverse nature of regeneration, understanding these processes will greatly benefit from a comparative/evolutionary analysis using a highly diverse subset of species.

NS regeneration is defined as the process by which damaged nervous tissue undergoes regrowth or renewal, restoring the system’s morphology and physiological functions. At the cellular level, NS regeneration can be divided into two independent processes: axonogenesis and neurogenesis. Axonogenesis is the process by which the axon of a pre-existing neuron regrows after suffering an injury, re-establishing the original connection. In vertebrates, axon regeneration is commonly seen in the peripheral nervous system (PNS); however, the same phenomenon is highly limited or fails to occur in the CNS. Axonogenesis does not fall within the scope of this review. For those interested in axonogenesis, we recommend some publications on the topic (Chisholm et al., 2016; Wahane et al., 2019).

Our focus is mainly on injury-induced neurogenesis and the mechanisms by which adult organisms activate neurogenesis in response to injury. Similar to the processes of cellular homeostasis or regeneration in other organs or tissues, new nerve cells originate from: (1) a reservoir of stem cells that can proliferate and differentiate to maintain tissue physiology or restore and repair an injury site or (2) by dedifferentiation, where a cell population reverts to a less-differentiated state by losing some of their differentiated properties (Tanaka, 2016; Yao and Wang, 2020; Walker and Echeverri, 2022). These cells can eventually divide and produce replacement cells for the organ or tissue in question. (3) A third mechanism has been proposed where tissues can generate new cells via trans-differentiation. In this case, similar to dedifferentiation, the cells lose their specific phenotypic characteristics and then acquire the phenotype of a different cell type without transiting through a “less differentiated stage” (Walker and Echeverri, 2022).

Regeneration is a dynamic process that can vary significantly from one species to another. A common mechanistic link to achieve NS regeneration has not been elucidated and, in fact, there might not be one common mechanism, but mechanisms might differ according to the animal groups. For example, protostomes with extensive regenerating abilities, such as Hydra or Planaria, are known to have pluripotent stem cells that can give rise to the NS cells and other cell types (Cebrià et al., 2002; Agata and Umesono, 2008). Crayfish, a well-studied crustacean, possess neurogenic niches with primary precursor neural progenitors (Sullivan and Beltz, 2005; Ventura et al., 2019). These cells can divide into secondary precursors that migrate to the olfactory structures and later differentiate into olfactory interneurons or projection neurons. The primary precursors cannot self-renew and depend on a re-supply of circulating hemocytes to replenish the precursor cell stock (Chaves da Silva et al., 2013; Benton et al., 2014). A comprehensive review on crustacean and insect adult neurogenesis and their regenerative mechanism can be found in Simões and Rhiner (2017).

## Radial glia-like cell role in adult vertebrate regeneration

In most vertebrates, CNS neuroregenerative capacities have been associated with the presence of RGLCs. These cells are well-known to repopulate and replenish injured tissues. Thus, organisms that retain RGLCs in their adult stages are associated with higher CNS regenerative abilities (Alvarez-Buylla et al., 1987; Malatesta et al., 2000; Malatesta and Götz, 2013; Hartfuss et al., 2001; Jurisch-Yaksi et al., 2020). For example, RGCs in rodents normally disappear soon after birth. In neonatal rodents whose brains have been injured, RGCs persist for a more extended period, suggesting that after neonatal brain injury, RGCs serve as scaffolds for neuroblasts to migrate and populate the lesioned site (Jinnou et al., 2018). Several studies have addressed the concept of proliferation and neurogenesis in adult rodents’ SVZ and SGZ after inducing focal ischemia or traumatic brain injury (Kernie and Parent, 2010). Following these injuries, progenitor cells on both niches respond by proliferating. Whether the cells generated by RGLC survive, migrate, and incorporate into CNS circuits remain controversial. Studies have determined that, in mammals, most of the cells generated in response to cerebral cortex injury do not survive and those that do are unable to successfully migrate to the lession site and differentiate (Chang et al., 2016). However, cell extrinsic factors have been shown to increase cell survivability and/or migration. For example, SVZ cell exposure to fibroblast growth factor (FGF2) increased cell survivability while inhibition of ADAM17 metalloprotease increased not only survival but also migration to the injured primary motor cortex (Chang et al., 2016; Geribaldi-Doldán et al., 2018).

While these studies expose the neurogenetic response to injury of adult RGLCs and their potential for facilitating CNS repair, these capacities in mammals are highly limited. Thus, the reason to focus on alternative model systems where the role of RGLCs in CNS regeneration can be explored extensively. Here we provide information on three of them, two vertebrate animals, the axolotl, the zebrafish, and an invertebrate, the sea cucumber.

## Regeneration in axolotl and zebrafish spinal cord

The spinal cord is a component of the CNS whose regenerative capacities have been intensively probed. While the formation of a glial scar hinders spinal cord regeneration in mammals, in some amphibians and fishes, morphological and functional recovery is possible. In the two best studied species, axolotl and zebrafish, new neurons are formed by so called “ependymal cells” surrounding the central spinal canal (Chernoff et al., 2003; Reimer et al., 2008). These ependymal cells clearly fulfill our description of RGLCs. First, they are derived from RGCs during development, Second, they retain their radial morphology and third they express some of the RG markers (GFAP and Sox2) (Hui et al., 2015b). In addition, several markers associated with RGCs are expressed by the proliferating cells following spinal cord injury (Hui et al., 2015a). After injury, these cells have been shown to retract their radial processes, proliferate and differentiate to form new spinal cord neurons (Reimer et al., 2008; Ribeiro et al., 2017; Cura Costa et al., 2021; Klatt Shaw et al., 2021; Walker and Echeverri, 2022). In fact, some researchers have suggested that these RGLCs undergo dedifferentiation during regeneration, as can be determined by the changes in cell morphology, the loss of the radial extensions, and the differential expression of genes associated with neural stem cell properties (Walder et al., 2003; Ribeiro et al., 2017; Walker and Echeverri, 2022).

It is important to contrast these RGLCs/ependymal cells of zebrafish and axolotl to those in adult mammals. In the latter, the ependymal cells are cuboidal and multiciliated and usually do not divide after differentiation or in some cases, give rise only to glia (Spassky et al., 2005; Meletis et al., 2008). Moreover, transcriptomic studies have shown that ependymal cells in mice are transcriptionally different from neural stem cells and are not induced to divide by brain injury (Shah et al., 2018).

Recent single-cell transcriptomics have identified distinct precursor populations that generate neuronal and glial cells during zebrafish embryonic development (Scott et al., 2021). Likewise, single cell transcriptomics have shown several populations of RGLCs to be present in the developing and adult axolotl brain, and one of these appears to be activated by injury (Wei et al., 2022). Thus, we expect that future experiments will provide crucial information on the ependymal populations in the adult zebrafish and axolotl and the transitions they undergo during spinal cord regeneration.

## Nervous system regeneration in zebrafish brain

Zebrafish RGLCs in different niches can generate new neurons under normal homeostatic conditions and in response to injury (Barbosa et al., 2004; Dray et al., 2015). However, there are differences between homeostatic neuron production and their formation in response to injury. In the former, newborn neurons remain below the progenitor zone while in the latter, newborn neurons migrate to populate injured brain regions (Barbosa et al., 2004; Dray et al., 2015). During normal conditions, only type II zebrafish RGLCs are in the active proliferative state (Dray et al., 2015). Their proliferation modes have been shown to be symmetric and asymmetric, providing self-renewing and progenitor-generating properties (Barbosa et al., 2004, Barbosa and Ninkovic, 2016; Rothenaigner et al., 2011; Dray et al., 2015). Most of the RGLC proliferation capacities are enhanced in response to injury. For example, using an engineered neurotoxic amyloid-β42 (Aβ42)-dependent zebrafish, which represents degenerative neurotoxicity similar to that observed in Alzheimer’s disease, researchers found that some RGLCs responded to this injury by extensive proliferation and enhanced neurogenesis (Bhattarai et al., 2016). One unique difference between homeostatic- and injury-generated neurons is that the latter have enhanced migratory capacities, rendering them able to restore damaged tissue (Barbosa et al., 2015; Barbosa and Ninkovic, 2016).

## Müller glia and regeneration of zebrafish retina

**FIGURE 6:**
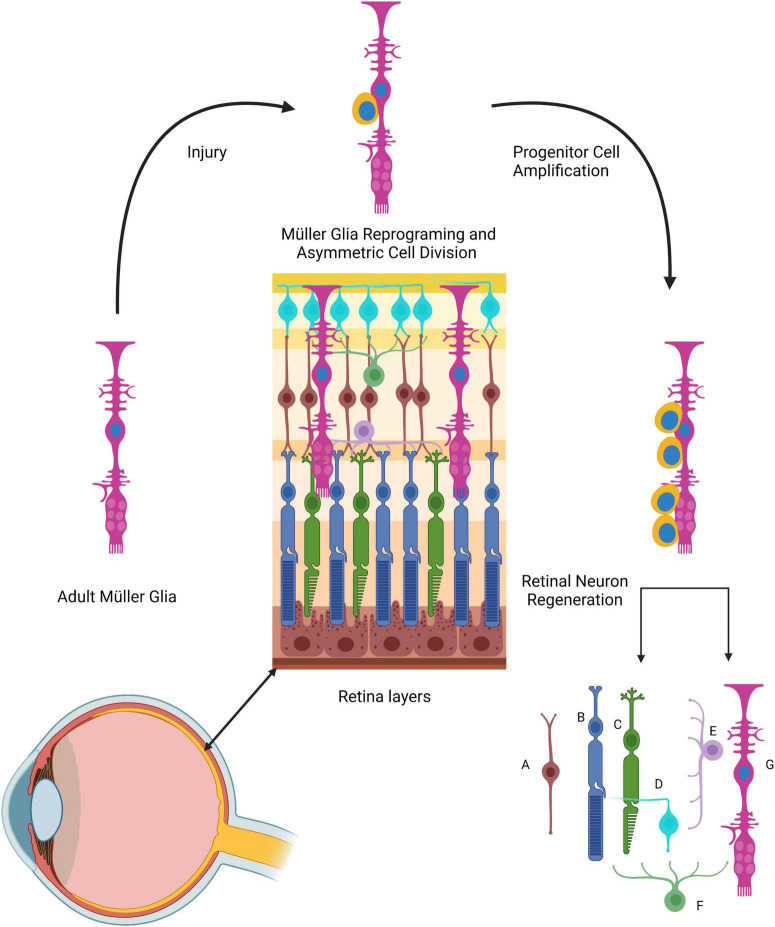
The role of Müller cells in zebrafish retinal regeneration. The Müller glia processes span across all retinal layers. Following injury, Müller cells (long magenta cells) dedifferentiate and acquire stem cell properties (and markers). These cells undergo asymmetric cell division, giving rise to round progenitor cells (yellow) that will form new Müller glia and Müller-derived progenitors that can generate all retinal cell types. (A) Retinal bipolar cell; (B) rod photoreceptor; (C) cone photoreceptor; (D) ganglion cell; (E) horizontal retinal cell; (F) amacrine cell; (G) Müller glia cell.

Zebrafish also display an impressive ability to regenerate their retina. The zebrafish retina, typical of vertebrate retinas, is composed of three cellular layers, six principal neuron population types, and four types of glial cells (Fadool and Dowling, 2008). One of the glial cells, the Müller glia, stands out in that it plays a unique role in retinal regeneration (Figure 6). The Müller glia apico-basal morphology enables structural and metabolic support for the retinal neuron population (Reichenbach and Bringmann, 2013; Lenkowski and Raymond, 2014). Müller glia are considered mature, differentiated cells. Under normal retinal physiology, they contribute to retinal synaptic activity by recycling GABA and glutamate neurotransmitters. Moreover, these cells support neurons by regulation of extracellular volume and molecular composition, transporting metabolic waste, and providing neurons with nutrients (Lenkowski and Raymond, 2014; Gao et al., 2021).

Müller glia have been proclaimed as being the retinal RGLC, mainly because of their radial morphology but also because they share some of the RGC markers, such as GS, GFAP, GLAST, and VIM (Linser and Moscona, 1979; Malchow et al., 1989; Lenkowski and Raymond, 2014; Gao et al., 2021). In response to injury, Müller glia play a pivotal role in retina regeneration. This process has been extensively studied at both cellular and molecular levels. It has been documented that these RGLCs respond to injury in the following manner: (i) Müller glia sense injury in the retina, (ii) The cells dedifferentiate or reprogram, (iii) Dedifferentiated Müller glia divide asymmetrically, producing one Müller glia cell and one Müller glia-derived progenitor cell, (iv) The progenitor cells divide and populate the injury site creating neurogenic clusters.

Transgenic zebrafish lines have provided crucial data to acknowledge Müller glia as the neural stem cell source for retinal regeneration (Bernardos and Raymond, 2006; Fimbel et al., 2007; Thummel et al., 2008). A study assessing their proliferation capacity by BrdU incorporation or PCNA immunodetection determined, that although there are proliferating Müller glia in the uninjured retina, most of the cells are in a quiescent state that is regulated by Notch signaling (Bernardos et al., 2007). As a response to injury, Notch expression decreases, facilitating Müller glia proliferation (Ruddy and Morshead, 2018; Sahu et al., 2021). Similar to other RGLCs, the Müller glia are also a source of neurons, as demonstrated by using transgenic reporter lines showing that proliferating Müller glia gave rise to rod progenitors (Bernardos et al., 2007).

Among the events that involve Müller glia in the regeneration process, we would like to highlight the dedifferentiation response since cell dedifferentiation stands out as a process by which RGLCs in various organisms appear to contribute to NS regeneration. Müller glia dedifferentiation has been assessed by the re-expression of retinal progenitor neurogenic markers, such as BLBP, ALCAMA, Rx1, six3b, and Pax6 (Fimbel et al., 2007; Thummel et al., 2008; Nagashima et al., 2013). In addition, Sox2 role has been functionally assessed in the regenerating zebrafish retina, demonstrating it to be an essential factor regulating Müller glia proliferation and reprogramming (Gorsuch et al., 2017; Elsaeidi et al., 2018). Even though progenitor marker re-expression is observed, it has been suggested that Müller glia cells only partially dedifferentiate, given that mature Müller glial cell markers persist at a lower level during dedifferentiation. Nonetheless, dedifferentiation enables Müller glia cells to divide asymmetrically producing a neural progenitor cell that proliferates and builds neurogenic clusters that will differentiate into the retina neural cell types (Bernardos et al., 2007).

Conserved functions of the Müller glia mediating ocular regeneration have also been documented in other vertebrates (Fischer and Bongini, 2010; Goldman, 2014). In the early 2000s, Fischer and Reh (2001) demonstrated that, in chickens, following retinal injury by injection of *N*-methyl-D-aspartate (NMDA), Müller glia cells re-entered the cell cycle and could differentiate into retinal neurons as determined by co-labeling of BrdU and retinal markers. The available data point to Müller glia cells as the essential cell for retinal repair (Fischer and Reh, 2003).

## Nervous system regeneration in echinoderms

**FIGURE 7:**
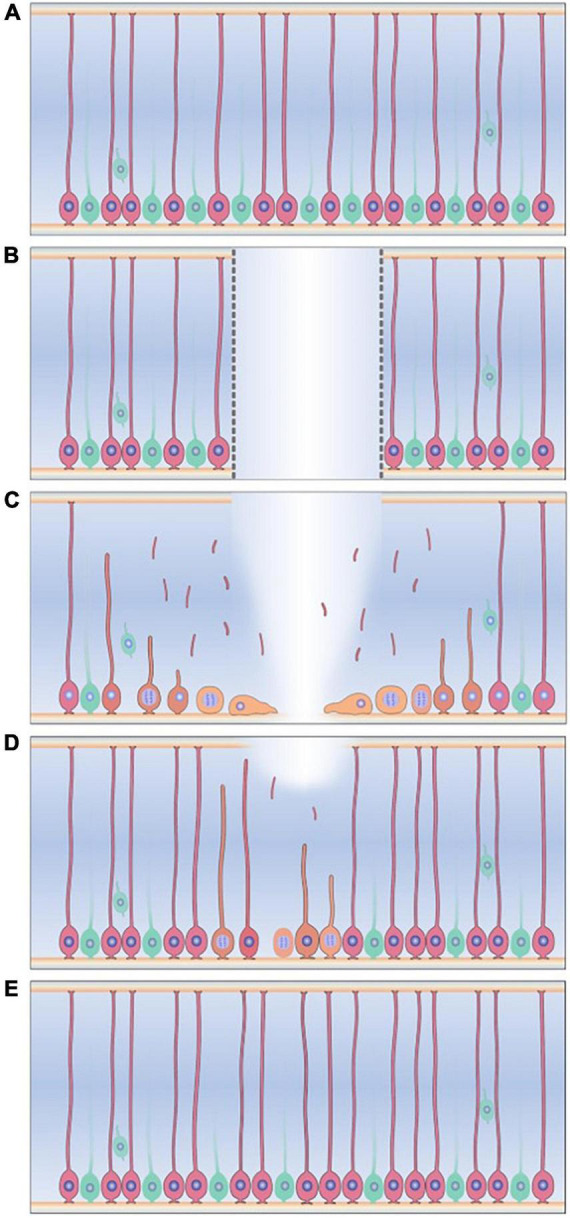
Radial glia-like cells response to injury in the sea cucumber *Holothuria glaberrima*. **(A)** Radial glia-like cells (pink) are the main glial cell found adjacent to neurons (green) in the holothurian radial nerve cord. **(B)** Transection of the radial nerve cord causes gap between two ends of the RNC **(C)** Radial glia-like cells adjacent to the injury dedifferentiate, as seen by degradation of cytoskeletal structures and loss of the radial processes. The dedifferentiated cells (yellow) are now able to proliferate and migrate to the injury gap. **(D)** The dedifferentiated cells and their progenitors give rise to the new radial glia-like cells and neurons that repopulate and restores the new tissue. **(E)** The regeneration process is completed when the progenitors of these cells differentiate into the new neurons and glia.

Radial glia-like cells also play a major role in the regeneration of the echinoderm NS following transection of the RNC. Immediately after transection, the injured edges of the RNCs begin to swell, nerve fibers become distorted, and programmed cell death occurs. This initial phase is characterized by extracellular matrix (ECM) deposition at the wound site and elongation of the RNC ectoneural component (Mashanov et al., 2008, 2014). At the cellular level, evidence using electron and fluorescence microscopy strongly suggests that the RGLCs dedifferentiate and provide the precursors for the new nervous tissue (Figure 7; San Miguel-Ruiz et al., 2009; Mashanov et al., 2010, 2013). Two observations have been key to outline the RGLCs role: First, the degradation of the RGLC radial cytoskeletal fibers during regeneration and second, BrdU experiments demonstrating the dedifferentiated RGLC proliferative capacities. The morphological changes are key features consistent with other known dedifferentiating cells. Hence, by losing their complex radial extensions, they transition into a less specialized state and proliferate. The dedifferentiated cells also migrate toward the injury gap and participate in one of the main events in RNC regeneration: the reconnection of the transected stumps. During this phase, both RNC edges from each side of the transected side close together until they reconnect and restore the RNC. This is thought to be one of the most complex processes involving signaling molecules that control growth, migration, axial polarity, and cell-to-cell interactions (San Miguel-Ruiz et al., 2009; Mashanov et al., 2013).

Following RNC reconnection, dedifferentiated cells in the regenerative zone redifferentiate, which is reflected by the growth in the size of the “new” tissue between the stumps. Birth dating experiments have shown that dedifferentiated RGLCs cells play a role in this event by giving rise to both RGLCs and neuronal populations in the regenerated structure (Mashanov et al., 2013). What regulates RGLC redifferentiation to neuronal or non-neuronal lineage still needs to be studied. However, many researchers have suggested that this process is mainly intrinsically regulated, while still having extrinsic signaling molecules and/or cell-to-cell interaction that could influence the outcome (Mashanov et al., 2008, 2013; San Miguel-Ruiz et al., 2009). By the end of the regenerative response, cell proliferation and apoptosis return to basal levels, and cellular and anatomical organization of the “new” RNC segment resembles that of the non-transected cords.

Much of what is known of RNC regeneration in holothurians has been provided by histological analyses, including immunohistochemistry, *in situ* RNA hybridizations, and RNA sequencing analysis. The transcriptomics data analyses have provided a list of differentially expressed genes that are possible candidates for genes controlling or modulating the regeneration response (Mashanov et al., 2014). For example, ECM-related genes were among the most differentially expressed transcripts in all regeneration stages. Many other genes have been targeted as possible candidates and even transposable elements have been suggested to be associated with RNC regeneration (Mashanov et al., 2012).

Specific genes have been probed to modulate particular cellular events, for example, the process of RGLC dedifferentiation. The first candidate genes to be evaluated as promoters of RGLC dedifferentiation were the Yamanaka pluripotency factors Oct1/2/11, c-Myc, Sox 2, and Klf1/2/4 homologs (Mashanov et al., 2015b). The study demonstrated that (i) all four factors were expressed during early and late stages of RNC regeneration, (ii) Myc was significantly overexpressed during all the stages of regeneration, suggesting that it played an important role in the regeneration process (iii) Oct1/2/11 and SoxB1 (sox 1 homolog) were significantly downregulated at the late stages of RNC regeneration (Mashanov et al., 2015b). These findings were further explored by evaluating the role of *Myc* via RNA knockdown in regenerating RNC. The results demonstrated that *Myc* regulated programmed cell death and RGLC cell dedifferentiation, two major events known to occur during the initial regeneration response (Mashanov et al., 2015c). Therefore, it has been assumed that the *in vivo* de-differentiation observed in the RGLCs is more akin to a partial reprogramming that partially dedifferentiates cells to a precursor-like phenotype than to the total reprogramming that produces pluripotential stem cells.

## New advances

Two recent advances open the possibility for new findings on the regeneration of the echinoderm CNS and the possible role of the RGLCs. The first is the development of an *in vitro* RNC explant preparation (Quesada-Díaz et al., 2021). Most, if not all, of the previously performed studies were done using the holothurian RNC complex, that in addition to the RNC is composed of longitudinal muscle bands, water vascular canal, and the body wall. This has made it almost impossible to directly target the RNC without affecting the surrounding tissues. Using an isolated RNC explant that can be cultured provides a unique opportunity to study cellular events associated with dedifferentiation and regeneration without the influence of adjacent tissues (Quesada-Díaz et al., 2021). Moreover, it has been documented that in *in vitro* cultures, RGLCs within the RNC continue to exhibit the same morphological changes shown to occur during *in vivo* RGC dedifferentiation. Hence, this method provides a tool to study the dedifferentiation process while excluding other cellular events in the surrounding tissues. This culture system is presently being used to provide a transcriptomic profile of regenerating RNC explants.

The second advancement with a significant impact on RNC regeneration studies is the sequencing of the *H. glaberrima* genome. This was published as a draft genome, providing a tool that can benefit both the regenerative and developmental biology fields (Medina-Feliciano et al., 2021). The genome serves as a database to identify potential genes and molecules that are key to the regeneration process. In addition, for researchers working with *H. glaberrima* that have obtained transcriptomic data from regenerating intestine and nerve complex at different time points, the genome provides a reference to the identification of the differentially-expressed genes. The impacts of these results will reach their full potential when a reference genome becomes available.

## Concluding remarks

Radial glial cells are found in all developing vertebrate CNS studied to date, and RGLCs are found in adult CNS components of vertebrates and invertebrates. The morphological data accumulated over decades, together with the avalanche of new molecular data provided by new sequencing methods is molding our view of these cells. In fact, we can describe the RG/RGLCs in terms of Ernst Haeckel dictum “Ontogeny recapitulates Phylogeny” where their evolution and their presence in different animal species parallels their embryological development. In this scenario, the RGCs that form in the early deuterostome embryo can be considered the precursors of both neurons and glia of the adult CNS. In invertebrate deuterostomes, these cells remain in the adult CNS, retaining morphological properties and particular molecular markers along with their competence to undergo neurogenesis. Thus, organisms where RGLCs are found are capable of robust CNS regeneration. A similar situation is observed in some amphibians and fishes, where the progeny of RGCs is retained as RGCLs and are responsible for both homeostatic and regenerative neurogenesis in adults. In contrast, in mammals, only certain areas of the adult CNS retain RGLCs. The cells in these niches might express specific markers along with more general RGLC markers, giving rise to particular subpopulations. Nonetheless, these RGLC subpopulations are able to undergo homeostatic neurogenesis and, after injury, these RGLCs are activated and appear to participate in some, albeit limited, CNS restoration. One last example will serve to strengthen the evolution/regeneration comparison. It is well documented that in most vertebrates, the capacity for CNS regeneration is lost during the developmental process. Nowhere is this better shown than in the Xenopus model where the ability to regenerate their transected spinal cord changes dramatically as the animal undergoes metamorphosis. In this respect, the work by the Larraín lab (Edwards-Faret et al., 2018, 2021) has correlated the regenerative capacity to the presence of RGLCs, expressing certain molecular markers and showing neurogenic capacity found in the regenerative stage but lost or greatly diminished in the non-regenerative stage.

In addition to the neurogenic regenerative potentials of RGLCs, there are also parallels in the processes by which RGLCs in different species or different regions of the CNS respond to injury. Specifically, changes observed in their radial morphology and, at the molecular level, in their gene expression profile point out toward a degree of dedifferentiation, where a mature cell reverts into a progenitor/stem cell phenotype as described by Walker and Echeverri (2022). Dedifferentiation allows cells to enter the cell cycle and give rise to new tissue cells. In this respect, it is interesting that activated astrocytes, following trauma to the adult mammalian brain, have also been shown to re-express markers of more immature precursors suggesting a degree of dedifferentiation (Zambusi et al., 2020; Lattke and Guillemot, 2022).

To fully understand the RGCs and RGLCs it becomes crucial to determine the lineage and evolutionary relationship of these cell types. Are all adult RGLCs remnants of the embryonic RGC population that persist in certain areas of the adult brain? Are the invertebrate RGLCs evolutionarily related to the vertebrate RGCs? What, if any, specific marker can be used to identify the RGLC and RGC subpopulations? Modern cell-typing techniques will be important in answering these and other questions. The use of gene expression markers will be decisive in determining the expression profile of the different cell types and the changes that might take place during developmental or functional stages. Equally important will be to determine the factors that induce the neurogenic activity in these cells and the process by which cells might dedifferentiate and/or enter the cell cycle.

The possibility exists that the neurogenic abilities of these cells can be manipulated to respond to traumatic injury so that they serve as proliferating neurogenic progenitors in favor of CNS repair. Apropos to this, dedifferentiation is also being explored as a potential therapy to regenerate the CNS. The idea is to be able to transform the state of the brain into a regeneration-promoting state by, for example, converting terminally differentiated astrocytes into a precursor type cell that could give rise to neurons (Yao and Wang, 2020; Zamboni et al., 2020; Robel et al., 2011; Lattke and Guillemot, 2022). Nonetheless, it is imperative to first lay a foundational understanding of RGC roles, their pathways of regulation, and possible differentiation stages. Key information could be obtained from comparative studies using non-traditional model animals that show extraordinary regeneration abilities. Therefore, we propose that the study of the RGLCs in the echinoderms, particularly in the sea cucumber *H. glaberrima* will provide important information to elucidate neurogenesis activation in adult animals.

## Author contributions

YM-N and JG-A wrote part of the manuscript, contributed to the article, and approved the submitted version.
